# Association between sleep quality and type 2 diabetes at 20-year follow-up in the Southall and Brent REvisited (SABRE) cohort: a triethnic analysis

**DOI:** 10.1136/jech-2020-215796

**Published:** 2021-06-11

**Authors:** Zhen Ling Ong, Nishi Chaturvedi, Therese Tillin, Caroline Dale, Victoria Garfield

**Affiliations:** 1 Department of Epidemiology and Public Health, Institute of Epidemiology and Health Care, University College London, London, UK; 2 MRC Unit for Lifelong Health and Ageing, Department of Population Science and Experimental Medicine, Institute of Cardiovascular Science, University College London, London, UK; 3 Department of Population Science and Experimental Medicine, Institute of Cardiovascular Science, University College London, London, UK; 4 Institute of Health Informatics, University College London, London, UK

**Keywords:** nutritional sciences, ethnic groups, sleep, longitudinal studies, epidemiology of diabetes

## Abstract

**Background:**

The risk of developing type 2 diabetes associated with poor sleep quality is comparable to other lifestyle factors (eg, overweight, physical inactivity). In the UK, these risk factors could not explain the two to three-fold excess risks in South-Asian and African-Caribbean men compared with Europeans. This study investigates (1) the association between mid-life sleep quality and later-life type 2 diabetes risk and (2) the potential modifying effect of ethnicity.

**Methods:**

The Southall and Brent REvisited cohort is composed of Europeans, South-Asians and African-Caribbeans (median follow-up 19 years). Complete-case analysis was performed on 2189 participants without diabetes at baseline (age=51.7±7 SD). Competing risks regressions were used to estimate the HRs of developing diabetes associated with self-reported baseline sleep (difficulty falling asleep, early morning waking, waking up tired, snoring and a composite sleep score), adjusting for confounders. Modifying effects of ethnicity were analysed by conducting interaction tests and ethnicity-stratified analyses.

**Results:**

There were 484 occurrences of incident type 2 diabetes (22%). Overall, there were no associations between sleep exposures and diabetes risk. Interaction tests suggested a possible modifying effect for South-Asians compared with Europeans for snoring only (p=0.056). The ethnicity-stratified analysis found an association with snoring among South-Asians (HR 1.41, 95% CI 1.08 to 1.85), comparing those who snored often/always versus occasionally/never. There were no elevated risks for the other sleep exposures.

**Conclusion:**

The association between snoring and type 2 diabetes appeared to be modified by ethnicity, and was strongest in South-Asians.

## Introduction

There is growing evidence for an association between sleep duration or quality and type 2 diabetes. Compared with sleep duration, fewer studies have explored the potential long-term effect of sleep quality on type 2 diabetes.^1^ While sleep duration refers to the number of hours asleep, sleep quality includes the subjective feeling of being rested, insomnia subtypes (eg, difficulty initiating sleep, difficulty maintaining sleep, early morning awakening), habitual snoring and other sleep-disordered breathing (SDB) that may lead to sleep fragmentation (ie, repetitive short awakenings).^2 3^

Evidence suggests that the relative risks of developing type 2 diabetes associated with difficulty initiating sleep, difficulty maintaining sleep and obstructive sleep apnoea (OSA) are 1.55, 1.72 and 1.49, respectively.^4^ OSA is a severe but relatively common form of SDB, characterised by loud snoring, breathing cessation and repeated nocturnal awakenings.^5^ These effect sizes are comparable to well-established type 2 diabetes risk factors, and only marginally smaller than having a family history of diabetes or overweight.^4^

Several possible mechanisms link poor sleep quality to impaired glucose metabolism and other cardiometabolic risks factors.^6 7^ In one study, three nights of laboratory-controlled sleep fragmentation without changing total sleep duration showed a 25% decreased insulin sensitivity^8^; comparable to a difference in type 2 diabetes risk associated with being 8–13 kg heavier.^9^ This suggests an adverse effect of poor sleep quality independent of total sleep duration. Another mechanism may involve energy imbalance through the upregulation of appetite (via decreased leptin and increased ghrelin)^10^ or reduced total energy expenditure due to day-time fatigue^11^ that predispose to weight gain and risk of type 2 diabetes.^6 7^ Other hypothesised pathways to impaired glycaemic control and insulin resistance involve increased evening cortisol levels and increased sympathetic tone which inhibits insulin release.^12^

Even fewer studies investigating the association between sleep quality and type 2 diabetes risk have included ethnic minorities,^5^ despite a twofold to threefold increased risk in some groups compared with Europeans.^13^ These excess risks appear only partially due to well-established biological and environmental risk factors.^13^ Given that poor sleep quality may be more prevalent among ethnic minorities,^14^ we hypothesised that there would be (1) a deleterious association between sleep quality measures (difficulty falling asleep, early morning waking, waking up feeling tired, and snoring)and type 2 diabetes risk and (2) stronger associations among South-Asians or African-Caribbeans.

## Methods

### Study design and population

Southall and Brent REvisited (SABRE) (2008–2012) is a multiethnic community-based prospective cohort composed of older Europeans, South-Asians and African-Caribbeans from London, set up to examine ethnic differences in cardiometabolic disorders. Median follow-up period was 19 (IQR=15–20) years. At baseline (1988–1991), there were more Europeans (48.8%) than South-Asians (38.8%) and African-Caribbeans (12.4%).^15^ Participants were between 40 and 69 years old and 75% were men.^13^ Participants’ ethnicity was initially determined by interviewers based on grand-parental origin and confirmed by participants.^13^ All South-Asians and African-Caribbeans were first-generation migrants. The African-Caribbeans originated from the Caribbean (91.5%) or West Africa.^13^ South-Asian participants originated from the Indian subcontinent, including Punjabi Sikhs (52%), Gujarati or Punjabi Hindus (20%), Muslims (15%) and other South-Asians (15%). Survivors at follow-up were between 57 and 90 years old. A detailed cohort profile and follow-up information are available elsewhere.^13 15^ The present study used data from baseline and 20-year follow-up.

### Measures

#### Exposure: sleep quality

At baseline, participants answered four questions on sleep quality, including whether they had difficulty falling asleep, woke up too early, felt tired on waking up and snored in the past 30 days. The first three questions were adapted from Jenkin’s Sleep Questionnaire,^16^ which is a brief, validated, reliable and widely-used sleep disturbance questionnaire. Responses were recoded as binary variables.

The snoring variable was dichotomised with ‘often’ and ‘almost always’ coded ‘yes’, whereas ‘occasionally’ and never’ were coded ‘no’. As one of the prominent symptoms for SDB, habitual snoring was used as a proxy marker. Although polysomnography is the gold standard diagnostic tool, questionnaires have previously been used to assess SDB prevalence.^17–19^

To summarise the multidimensional nature of sleep health,^2^ we constructed a composite measure of overall sleep quality from a principal component analysis using the methodology from Topriceanu *et al*.^20^ This score was a weighted average of sleep exposures using the first principal weights, standardised with mean=0 and SD=1.

#### Outcome: incident type 2 diabetes

Because sleep disturbances may be a symptom of type 2 diabetes, participants with diabetes at baseline were excluded to ensure that sleep quality exposures preceded any type 2 diabetes outcome. Baseline type 2 diabetes was ascertained by (1) self-report of doctor diagnosis or (2) receipt of antidiabetes medications (3) fasting blood glucose ≥7.0 mmol/L or postload glucose after an oral glucose tolerance test (OGTT) ≥11 mmol/L following WHO criteria.^21^

For incident type 2 diabetes, participants were directly followed up using (1) primary care medical record review of diagnosis or prescribed antidiabetic medication, (2) self-completion questionnaires of physician diagnosis with year of diagnosis or named antidiabetic medication and (3) OGTT ≥11 mmol/L at follow-up.^15^

Participants who had died due to type 2 diabetes between baseline and follow-up but did not have a recorded year of diagnosis were excluded from the analysis, as this information was necessary for competing risks regression modelling.

#### Covariates

Several baseline measured covariates were adjusted for in three hierarchical models. Model 1 controlled for demographic factors (age, sex, ethnicity and socioeconomic position (SEP) measured by years of education); model 2 additionally adjusted for self-reported health behaviours (smoking status, physical activity); model 3 additionally adjusted for general adiposity (body mass index, BMI). These covariates were established a priori risk factors for type 2 diabetes^13 22^ and also associated with sleep. Our study controlled for general adiposity instead of central adiposity (using waist-to-hip ratio) as general adiposity may be more important in relation to sleep quality, particularly for OSA.^23^

### Statistical analyses

Statistical analyses were carried out using RStudio V.1.1.456 and Stata V.16.

**Figure 1:**
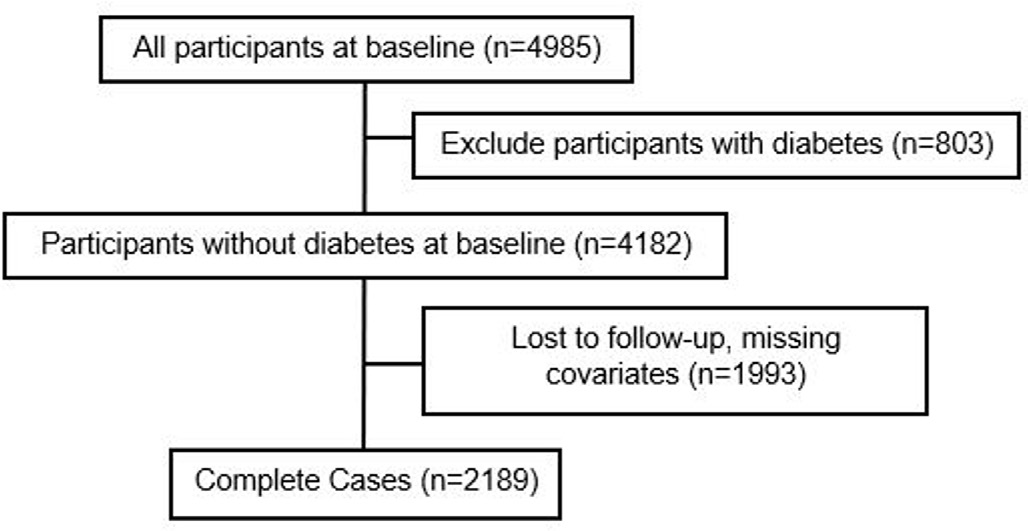
Flow chart to obtain the complete case sample (n=2189).

This study used a complete-case analysis (CCA) (figure 1, n=2189) to ensure comparability across different models. From a baseline of 4985 participants, this study excluded participants with prevalent diabetes (n=803), those lost to follow-up (LTFU) for diabetes status, year of censoring for incident diabetes and year of censoring for all-cause mortality and participants with missing data on any covariates (n=1993).

### Descriptive statistics

Baseline characteristics were presented for all covariates, grouped by incident type 2 diabetes status and ethnicity. Results are expressed as geometric mean±SD for continuous variables and frequency with percentages for categorical variables. All formal tests of differences between groups were conducted using one-way analysis of variance for continuous variables and Pearson’s χ^2^ tests for categorical variables. The patterns of sleep quality exposures were also examined by ethnicity and incident type 2 diabetes status.

### Competing risks regression

Competing risks hazard regression models were used to calculate (sub)HRs and 95% CIs of developing type 2 diabetes for the four sleep quality exposures specified within the same model. Those who reported no sleep problems formed the reference group.

The outcome for the survival model was time from baseline until the development of type 2 diabetes or censoring. Death from other causes than type 2 diabetes was considered as a competing event, as a previous investigation in SABRE supports ethnic differences in the association between sleep quality and various causes of mortality: early morning waking seemed to increase the risk of CVD mortality among Europeans, while difficulty falling asleep seemed to moderately increase the risk of all-cause mortality in South-Asians.^24^ Participants without death notification were censored at the end of the follow-up time.

The three hierarchical competing risk models were specified and fitted in the whole analytical sample. We checked the proportional hazards assumption in all models by including time-varying covariates.

### Modifying effects of ethnicity

The potential modifying effects of ethnicity on the association between sleep quality and incident type 2 diabetes were explored by adding four interaction terms for each ethnicity*sleep quality exposure in the fully adjusted model (model 3). The Europeans formed the reference group. Any interaction terms below the alpha threshold of 0.10 were taken forward by running the models within ethnicity-stratified samples using an alpha of 0.05.

### Sensitivity analysis

A single sleep assessment at baseline may not capture changes over the long follow-up period, thus, we analysed the risk of incident diabetes in the first-half vs the second-half of follow-up (ie, ≤10 years or >10 years of follow-up) by testing for interactions (follow-up period*sleep variable), followed by a stratified analysis of each time periods.

### Attrition

Over one-third of participants without diabetes at baseline were LTFU despite extensive efforts for tracing and follow-up. Missingness on all other covariates was negligible (<7%). Baseline characteristics of those LTFU or had missing covariates were compared with the CCA sample.

## Results

### Baseline characteristics

Table 1 shows the baseline characteristic of participants stratified by ethnicity and incident diabetes status. Among the 2189 complete cases, 484 people (22%) had developed type 2 diabetes at 20-year follow-up. Only 14% of the Europeans developed type 2 diabetes, compared with 33% of South-Asians and 30% of African-Caribbeans. Type 2 diabetes incidence was associated with nearly all covariates, except for SEP.

**Table 1:** Baseline characteristics by incident type 2 diabetes (T2D) status and ethnicity, complete cases (N=2189)

Baseline characteristics	Incident T2D (N=484)				No T2D (N=1705)			
	EU (N=168)	SA (N=232)	AC (N=84)	P value	EU (N=1031)	SA (N=473)	AC (N=201)	P value
Age (years)	51.5 (6.4)	49.4 (6.2)	53.1 (5.7)	0.489	52.9 (7.2)	50.3 (6.9)	52.8 (6.3)	0.003**
Sex (proportion male)	132 (79)	212 (91)	50 (60)	<0.001***	802 (78)	**393** (**83**)	**114** (**57**)	<0.001***
SEP (years of education)	10.4 (2.2)	12.3 (3.6)	10.8 (2.7)	0.032*	10.7 (2.6)	12.4 (3.8)	11.0 (3.0)	<0.001***
Physical activity (MJ/wk)	10.7 (6.7)	9.4 (5.9)	10.8 (7.7)	0.664	11.6 (7.3)	**9.8** (**6.8**)	**11.4** (**6.7**)	0.015*
Smoking status				<0.001***				<0.001***
Never smoker	53 (32)	161 (69)	60 (71)		325 (32)	383 (81)	129 (64)	
Ex-smoker	59 (35)	45 (19)	13 (15)		326 (32)	49 (10)	40 (20)	
Current smoker	56 (33)	26 (11)	11 (13)		380 (37)	41 (9)	32 (16)	
BMI (kg/m^2^)	28.3 (4.7)	26.7 (3.2)	29.0 (4.8)	0.922	25.5 (3.6)	25.4 (3.7)	26.9 (4.2)	<0.001***

Values represent means (SDs) or n (%).

P value from either one-way ANOVA or Pearson’s χ^2^ test. P:***<0.001, **<0.01, *<0.05. Bold P values represent P<0.05.

AC, African-Caribbeans; ANOVA, analysis of variance; BMI, body mass index; EU, Europeans; SA, South-Asians; SEP, socioeconomic position.

### Pattern of sleep quality exposures

**Figure 2:**
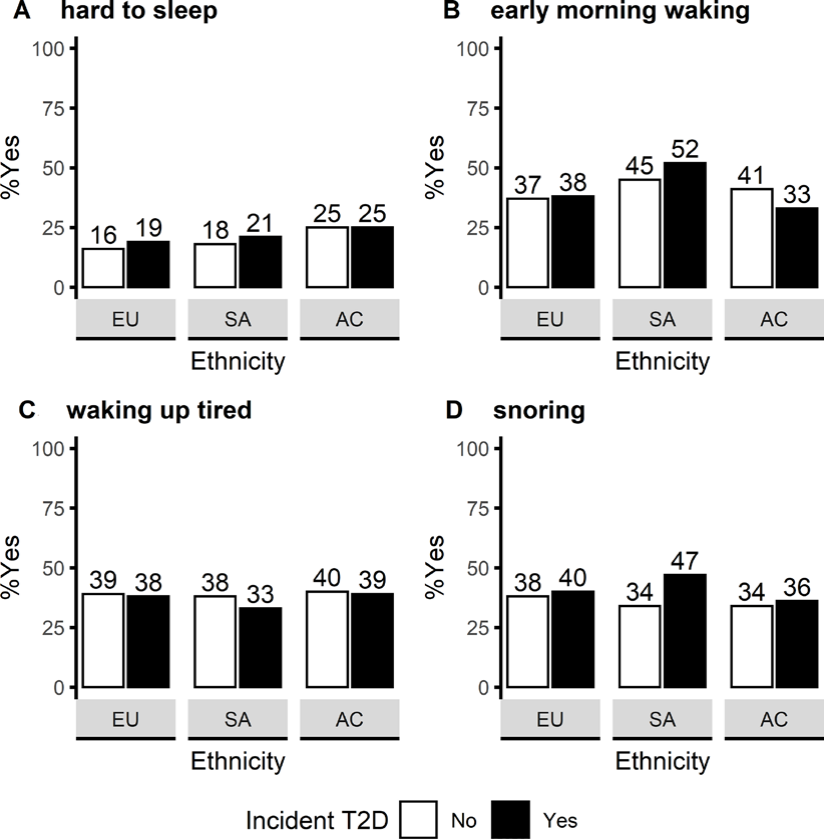
Prevalence of sleep problems by ethnicity and incident T2D status. Y-axis represents the percentage of ‘yes’ respondents for the sleep quality exposures. Sample sizes (% of complete case sample): (A) n=406 (19%); (B) n=887 (41%); (C) n=834 (38%); (D) n=823 (38%). AC, African-Caribbeans; EU, Europeans; SA, South-Asians; T2D, type 2 diabetes.

Early morning waking was the most commonly reported sleep problem (41%), followed by tiredness on waking (38%), snoring (38%) and difficulty falling asleep (19%). For all ethnicities combined, snoring prevalence was found to be different between people who developed type 2 diabetes and those who did not (43% and 36%, respectively). This difference seemed to be driven by snoring patterns among South-Asians (figure 2). Snoring was reported by 47% of South-Asians who developed type 2 diabetes, in contrast with 34% by people who did not develop type 2 diabetes. Interethnic differences were also found for difficulty sleeping and early morning waking; African-Caribbeans tended to have greater difficulty falling asleep; South-Asians tended to wake up too early.

### Competing risks regression

Table 2 presents the HRs and 95% CIs for the risk of developing type 2 diabetes at 20-year follow-up, as well as the number of participants who developed type-2 diabetes and person-years followed-up in the exposed and reference groups. For the individual sleep exposures, we confirmed that the proportional hazards assumption was met and therefore present results from the models without time-varying covariates. In the overall sample, there was some evidence for an association between snoring and type 2 diabetes (models 1 and 2) but this attenuated to the null when general adiposity was included in the model. No association was found for the composite sleep quality score either.

**Table 2:** Overall and ethnicity-stratified analysis reported in HRs (95% CI), complete case analysis (n=2189)

Sleep variable	Number of type-2 diabetes occurrences and person-years followed up (Cases/P-Y)		Model 1	Model 2	Model 3	P-value for interaction
Difficulty Falling Asleep	No	Yes				
All	383/30 741	101/6631	1.21 (0.96 to 1.54)	1.18 (0.93 to 1.50)	1.21 (0.95 to 1.54)	
Europeans	136/17591	32/3324	1.26 (0.84 to 1.89)	1.25 (0.83 to 1.89)	1.26 (0.83 to 1.93)	–
South-Asians	184/9708	48/2216	1.28 (0.91 to 1.79)	1.12 (0.80 to 1.59)	1.16 (0.82 to 1.65)	0.748
African-Caribbeans	63/3442	21/1091	1.16 (0.65 to 2.10)	1.18 (0.64 to 2.17)	1.12 (0.62 to 1.99)	0.786
Early Morning Waking	No	Yes				
All	272/22 367	212/15 005	1.12 (0.93 to 1.35)	1.14 (0.94 to 1.38)	1.11 (0.92 to 1.34)	
Europeans	104/13 291	64/7624	1.04 (0.75 to 1.44)	1.04 (0.75 to 1.44)	1.00 (0.72 to 1.40)	–
South-Asians	112/6294	120/5630	1.17 (0.90 to 1.53)	1.21 (0.93 to 1.58)	1.21 (0.92 to 1.58)	0.392
African-Caribbeans	56/2782	28/1751	0.67 (0.38 to 1.19)	0.68 (0.38 to 1.21)	0.60 (0.33 to 1.09)	0.133
Waking up Tired	No	Yes				
All	301/23 096	174/14 276	0.82 (0.68 to 1.01)	0.80 (0.66 to 0.98)	0.80 (0.66 to 0.98)	
Europeans	104/12 757	64/8158	0.88 (0.62 to 1.21)	0.84 (0.60 to 1.17)	0.87 (0.62 to 1.22)	–
South-Asians	155/7558	77/4366	**0.75 (0.56 to 1.00**)	**0.71 (0.53 to 0.95**)	**0.72 (0.54 to 0.97**)	0.389
African-Caribbeans	51/2781	33/1752	1.16 (0.71 to 1.91)	1.18 (0.72 to 1.94)	1.14 (0.68 to 1.92)	0.607
Snoring	No	Yes				
All	277/13 985	207/23 387	1.33 (1.10 to 1.60)	1.32 (1.10 to 1.59)	1.15 (0.96 to 1.39)	
Europeans	100/7974	68/12 941	1.15 (0.83 to 1.58)	1.12 (0.82 to 1.54)	0.91 (0.67 to 1.23)	–
South-Asians	123/4479	109/7445	**1.53 (1.17 to 1.98**)	**1.56 (1.20 to 2.03**)	**1.41 (1.08 to 1.85**)	**0.056**
African-Caribbeans	54/1532	30/3001	1.07 (0.68 to 1.68)	1.09 (0.69 to 1.72)	0.93 (0.59 to 1.46)	0.945

Model 1: adjusted for age, sex and ethnicity, socioeconomic position; model 2: model 1 plus physical activity and smoking status; model 3: model 2 plus BMI; P=p values for interaction by ethnicity (ethnicity*sleep quality exposure), alpha threshold=0.10, Europeans as the reference group. Bold values represent HR and 95%CI that do not cross 1.00 or P values for interaction by ethnicity <0.10.

BMI, body mass index; P-Y, person-years followed up.

### Modifying effects of ethnicity

The interaction effect models suggested a potential modifying effect of being of South-Asian origin compared with Europeans for snoring only (p=0.056).

In the ethnicity-stratified analysis (table 2), a distinctly stronger association for snoring was found for South-Asians (HR 1.41, 95% CI 1.08 to 1.85) (model 3). There was no evidence for this association among Europeans or African-Caribbeans in any models. There was a relatively strong negative association between waking up tired and incidence of type 2 diabetes (0.72, 0.54–0.97) (model 3) among South-Asians.

For difficulty falling asleep and early morning waking, there were no apparent associations with type 2 diabetes risk across all models. For the overall sleep quality measure, the proportional hazards assumption was not met in the South-Asian subsample but no associations with type 2 diabetes were found across all models. Please refer to online electronic supplemental material 1.

10.1136/jech-2020-215796.supp1 Supplementary data

### Sensitivity analysis

There was no strong evidence of an interaction with length of follow-up for snoring, difficulty falling asleep and waking up tired. There was a strong interaction for early morning waking (p=0.004); the stratified analysis by the two time periods (≤10 years or >10 years) suggested a stronger association with type 2 diabetes risk in the later period. Results stratified by follow-up period are presented in online electronic supplemental material 2.

## Discussion

Results show that snoring was associated with incident type 2 diabetes, but was observed only in South-Asians and not in Europeans or African-Caribbeans. Difficulty falling asleep, early morning waking, feeling tired on waking up, and the composite sleep quality measure were not associated with type 2 diabetes risk across all ethnic groups.

The association found for snoring among South-Asians is comparable to the pooled unadjusted relative risk for OSA in the same meta-analysis,^4^ which is striking considering OSA is a severe form of SDB The two-fold increased type 2 diabetes risk associated with snoring observed only in South-Asians represents a novel finding. To extend previous work on ethnic differentials in type 2 diabetes risk,^13^ our findings suggest that snoring could play a more prominent role in type 2 diabetes pathogenesis in South-Asians. Our study lacked objectively diagnosed SDB (including OSA). Nevertheless, previous studies show that the progression of increased apnoea/hypopnoea index (indicating OSA presence and severity) among primary snorers and untreated patients with mild-to-moderate OSA were significantly associated with length of time.^25^

For OSA specifically, hypothesised mechanisms involve sleep fragmentation, intermittent hypoxia (periodic deoxygenation and reoxygenation of blood due to recurrent airway blockage), and reduced total sleep duration. These states may predispose to glucose intolerance and insulin resistance through systemic inflammation and increased oxidative stress.^26^ As a proxy for SDB, it is possible that frequent snoring may share some of these mechanisms in South-Asians.

The association between snoring and type 2 diabetes risk independent of adiposity was consistent with findings from a meta-analysis that included OSA.^27^ Obesity is a prominent shared risk factor for OSA and type 2 diabetes. Fat deposition surrounding the neck, chest and abdomen may compromise airway space and collapsibility, predisposing individuals to OSA.^28^ Yet, frequent snoring has also been independently associated with glucose intolerance in lean adults.^29^

Our null findings for difficulty falling asleep and difficulty maintaining sleep (measured as ‘early morning waking’) were not comparable with a recent meta-analysis.^4^ Study-specific characteristics may explain these results. Studies included in the meta-analysis typically involved ethnically homogenous groups from wealthy countries. Although most of these studies included similar self-reported sleep quality questions, the accuracy of self-reported sleep has been found to vary by ethnicity due to systematic differences in the perception and reporting of sleep measures.^30^ This may partly contribute to the observed heterogeneity by ethnicity in this study. Moreover, Jenkin’s Sleep scale has not been cross-culturally validated. These questions may be more appropriate for Western monophasic sleep cultures (one sleep session at night) but less suited for the siesta sleep culture (daytime napping and nocturnal sleep) common in Caribbean communities.^31^ Future sleep research should use cross-culturally validated sleep questionnaires or multigroup analysis to ensure measurement invariance across ethnic groups.

Our study had several limitations. Using a single self-reported sleep assessment that has not been compared against objective measures (eg, polysomnography) may have attenuated the observed associations due to measurement error. Other studies that used questionnaires to measure snoring frequency as a proxy for SDB typically also include other SDB or OSA symptoms (eg, snoring intensity, apnoea-like symptoms and daytime sleepiness).^17–19^ Using sleep quality data at one time point in 20 years do not allow us to capture the cumulative exposures and changes over time. Ageing is associated with decreased sleep quantity and quality,^32^ and more day-time napping.^33^ Retirement may have changed participants’ sleep quality if sleep disturbances such as insomnia were work-related (eg, work-related stressors). More free time post-retirement may also allow for longer sleep or napping to compensate for poor sleep quality. Indeed, laboratory experiments have found that recovery sleep following a time of restricted sleep could partially reverse disruption in glucose metabolism over the short term.^34^ For migrant groups, poor sleep quality caused by acculturative stress might also improve over time on successful integration to the host society.^35^ We attempted to address some of these limitations through the sensitivity analysis, which found a stronger association for early morning waking in the later period. Early waking in mid-life, presumably for work, could perhaps have a protective effect related to higher SEPs. Future research should use repeated measures to characterise trajectories of sleep quality alongside changes in sleep duration.^36 37^

Another limitation of observational studies includes residual confounding, particularly for family history of diabetes and dietary patterns (eg, hypercaloric diets, caffeine intake).^38^ Other unmeasured aspects of SEP (eg, wealth), migration-related health determinants and adverse childhood exposures may also explain observed ethnic differences.^39^ Furthermore, attrition may introduce bias but it is difficult to ascertain the direction of bias. Certain differences between the CCA and LTFU sample may contribute to overestimation (ie, more males, fewer never-smokers and more ex-smokers in the CCA sample) whereas others may contribute to underestimation (ie, more Europeans, fewer African-Caribbeans, more years of education and slightly lower BMI in the CCA sample) online electronic supplemental material 3.

Given the comparable effect size of poor sleep quality with well-established type 2 diabetes risk factors,^4^ identifying cost-effective and scalable early interventions to optimise sleep quality and duration show potential for type 2 diabetes prevention. While outside the scope of this study, there is also value in exploring sleep interventions for the management of type 2 diabetes. In a systematic review of clinical practice guidelines (CPG) to identify sleep recommendations for type 2 diabetes management,^40^ 86% of the CPGs identified suboptimal sleep as potentially causing or contributing to diabetic comorbidities but few discussed sleep as a therapeutic target.

In conclusion, our findings suggest that sleep quality, particularly snoring in the middle age is associated with the development of type 2 diabetes in later life, even after adjusting for well-established type 2 diabetes risk factors. This association was only found in South-Asians but not among the Europeans and African-Caribbeans. Findings do not suggest significant associations for difficulty falling asleep, early morning waking and waking up tired. This warrants further investigation into the apparent ethnic inequality and any sources of resilience due to changes in sleep pattern postretirement. This study may inform strategies for type 2 diabetes prevention among South-Asians through screening for frequent snoring, SDB or OSA as risk factors for type 2 diabetes.

**What is already known on this subject**

- Poor sleep quality (difficulty falling asleep, difficulty maintaining sleep and sleep-disordered breathing) is associated with increased risk of developing type 2 diabetes.
- Current literature lacks evidence for ethnically diverse cohorts with lengthy follow-up.

**What this study adds**

- Findings suggested that snoring (proxy for sleep-disordered breathing) was associated with incident type 2 diabetes, observed only in South-Asians but not in Europeans and African-Caribbeans.
- Future studies should identify causes of this apparent ethnic inequality by considering changes in sleep quality after retirement and other aspects of sleep (eg, duration, timing).
- Opportunities to reduce ethnic-health inequalities in type 2 diabetes risk include screening for snoring as a risk factor among South-Asians and examining whether available treatments may reverse this risk.

## Data Availability

Data are available on reasonable request. Data may be obtained from a third party and are not publicly available. The dataset analysed are available from the SABRE Study Group but restrictions apply to the availability of these data, which were used with permission for the current study, and so are not publicly available. Data are however available from the corresponding author upon reasonable request and with permission from the SABRE Study Group.
