# Supplementary material for: Association between sleep quality and type 2 diabetes at 20-year follow-up in the Southall and Brent REvisited (SABRE) cohort: a triethnic analysis
Source: J Epidemiol Community Health. 2021 Jun 11;75(11):1117–22. doi: 10.1136/jech-2020-215796 (PMC8515117; doi:10.1136/jech-2020-215796)
Supplement: Supplementary data [file jech-2020-215796supp001.pdf]

Electronic Supplementary Materials (ESM)

ESM-1— Overall and ethnicity-stratified analysis of the association between the composite sleep quality variable and type-2 diabetes risk reported in hazard ratios (95%CI), complete case analysis (n=2189)

Sleep variable	Person-years followed-up			Hazard ratio			
z_wSleep	Type-2 diabetes		Total	Model 1	Model 2	Model 3	P-value for interaction
	No	Yes					
All	30863	6509	37372	1.03 (0.94-1.13)	1.02 (0.93-1.11)	1.00 (0.92-1.10)	-
Europeans	18488	2427	20915	1.04 (0.88-1.22)	1.02 (0.87-1.20)	1.01 (0.86-1.18)	-
South-Asians	8862	3062	11924	1.06 (0.94-1.20)	1.02 (0.90-1.16)	1.03 (0.90-1.17)	0.980
African-Caribbeans	3513	1020	4533	0.95 (0.78-1.15)	0.96 (0.79-1.16)	0.89 (0.73-1.09)	0.209

Model 1: adjusted for age, sex, and ethnicity, socioeconomic position; **Model 2**: model 1 plus physical activity, and smoking status; **Model 3**: model 2 plus BMI; z_wSleep= standardised, composite wSleep score; P= p-values for interaction by ethnicity (ethnicity*sleep quality exposure), alpha threshold=0.10, Europeans as the reference group

ESM-2—Sensitivity analysis stratified by follow-up period (≤10 years or >10 years)

Sleep Variable	≤10 Years Follow-up	>10 Years Follow-up	P-value for interaction
**Difficulty Falling Asleep**			
Model 1	1.15 (0.72-1.82)	1.28 (0.97-1.68)	0.122
Model 2	1.12 (0.70-1.78)	1.24 (0.94-1.63)	0.116
Model 3	1.09 (0.68-1.75)	1.29 (0.98-1.71)	0.074
**Early Morning Waking**			
Model 1	0.92 (0.64-1.32)	1.19 (0.95-1.49)	**0.005**
Model 2	0.94 (0.65-1.34)	1.22 (0.97-1.53)	**0.004**
Model 3	0.93 (0.65-1.33)	1.18 (0.94-1.48)	**0.004**
**Waking up Tired**			
Model 1	1.07 (0.74-1.55)	**0.75 (0.60-0.95)**	0.575
Model 2	1.04 (0.71-1.51)	**0.73 (0.58-0.92)**	0.577
Model 3	1.04 (0.70-1.53)	**0.73 (0.58-0.92)**	0.482
**Snoring**			
Model 1	**1.44 (1.02-2.03)**	**1.25 (1.00-1.55)**	0.282
Model 2	**1.43 (1.02-2.02)**	1.23 (0.99-1.54)	0.272
Model 3	1.22 (0.86-1.72)	1.08 (0.87-1.35)	0.265

Model 1: adjusted for age, sex, and ethnicity, socioeconomic position; **Model 2**: model 1 plus physical activity, and smoking status; **Model 3**: model 2 plus BMI; P= p-values for interaction by follow-up period (*follow-up period*sleep quality exposure*)

ESM-3—Baseline characteristics of participants lost to follow-up (LTFU or had missing covariates (N=1993) and complete cases (N=2189)

	LTFU or missing covariates (N = 1993)	Complete cases (N = 2189)	P
**Age at Baseline**	52.2 (6.9)	51.9 (7.0)	0.073
**Sex (Proportion male)**	1,424 (71)	1703 (78)	0.001***
**Ethnicity:**			<0.001***
**Europeans**	992 (50)	1,199 (55)	
**South-Asians**	639 (32)	705 (32)	
**African-Carribeans,**	362 (18)	285 (13)	
**SEP (Years of education)**	10.8 (3.1)	11.3 (3.1)	<0.001***
**Physical Activity Score (MJ/wk)**	10.6 (7.3)	10.9 (7.0)	0.243
**Smoking Status**			0.035*
**Never smoker**	1,062 (54)	1,111 (51)	
**Ex-smoker**	428 (22)	532 (24)	
**Current smoker**	493 (25)	546 (25)	
**BMI**	26.4 (4.0)	26.1 (3.9)	0.002**

Values represent means (SDs) or n (%), BMI = body mass index, SEP= socioeconomic position. Number of missing variables for LTFU or missing covariates group: Age at baseline(n=4); SEP(n=144); Smoking Status(n=10); BMI(n=3), P = p-value from either one-way ANOVA or Pearson's chi-squared test.
